# 569. Identification of Ampicillin and Vancomycin In Vitro Susceptibility Test Interpretive Criteria for Enterococcus faecalis and Enterococcus faecium

**DOI:** 10.1093/ofid/ofaf695.178

**Published:** 2026-01-11

**Authors:** Sujata M Bhavnani, Jeffrey P Hammel, Christopher M Rubino, Brian D VanScoy, M Courtney Safir, Jennifer K Torgersen, Rodrigo E Mendes, Helio Sader, Paul G Ambrose

**Affiliations:** Institute for Clinical Pharmacodynamics, Schenectady, New York; Institute for Clinical Pharmacodynamics, Schenectady, New York; Institute for Clinical Pharmacodynamics, Schenectady, New York; Institute for Clinical Pharmacodynamics, Schenectady, New York; Institute for Clinical Pharmacodynamics, Schenectady, New York; Institute for Clinical Pharmacodynamics, Inc., Schenectady, New York; Element Iowa City (JMI Laboratories), North Liberty, IA; Institute for Clinical Pharmacodynamics, Schenectady, New York

## Abstract

**Background:**

Ampicillin and vancomycin are used for the treatment of patients with enterococcal infections. *In vitro* STIC for these agents against *Enterococcus* species were established decades ago based on limited clinical and pharmacokinetic-pharmacodynamic (PK-PD) data. Using non-clinical PK-PD targets for efficacy, population PK models, simulation, and *in vitro* surveillance data, PK-PD target attainment analyses were performed to identify STIC for these agents against *E. faecalis* and *E. faecium*.

**Figure:**
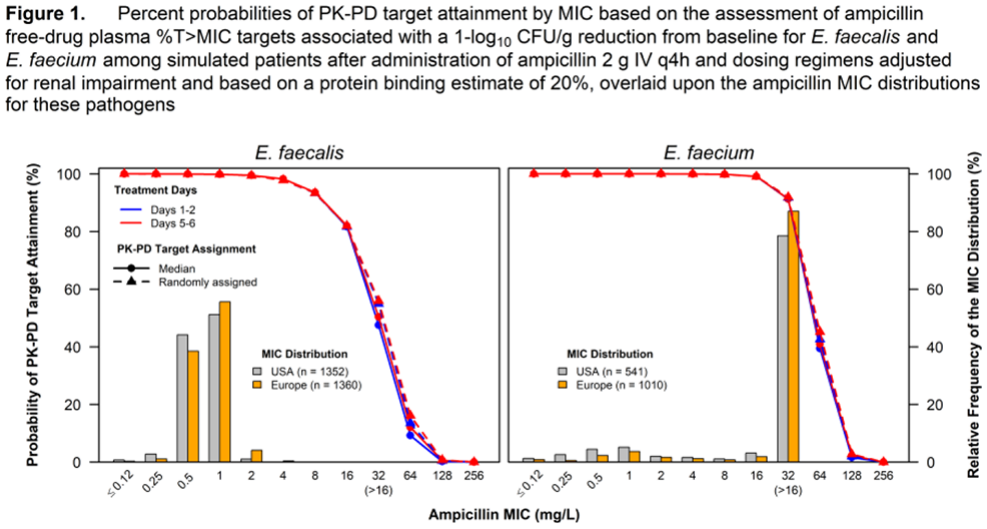

**Figure:**
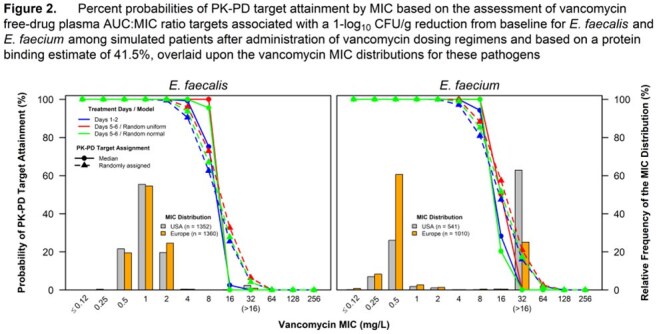

**Methods:**

Population PK models for each agent were identified from the literature. PK-PD targets for efficacy were based on data from a neutropenic murine invasive enterococcal infection model. Using replication, simulated patients with demographic variables resembling a clinical trial population were generated. These variables with population PK models were used to generate ampicillin and vancomycin exposures after administration of ampicillin 2 g IV q4h adjusted for creatinine clearance (CLcr) and vancomycin weight-based loading doses followed by dosing regimens based on weight and CLcr. On Days 5 to 6, vancomycin AUC values were assigned from distributions assuming administration of dosing regimens adjusted using therapeutic drug monitoring. Percent probabilities of PK-PD target attainment by MIC based on median and randomly assigned ampicillin and vancomycin PK-PD targets associated with a 1-log_10_ CFU reduction from baseline for each pathogen were assessed in the context of *in vitro* surveillance data and any available clinical PK-PD and outcome by MIC data from the literature.

**Figure:**
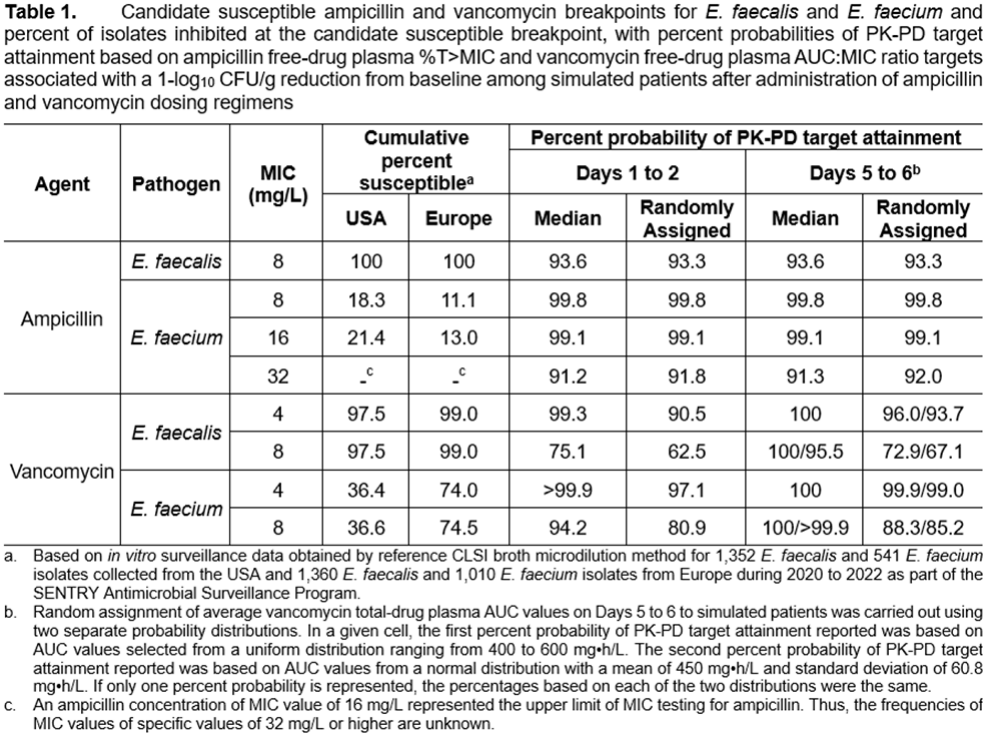

**Figure:**
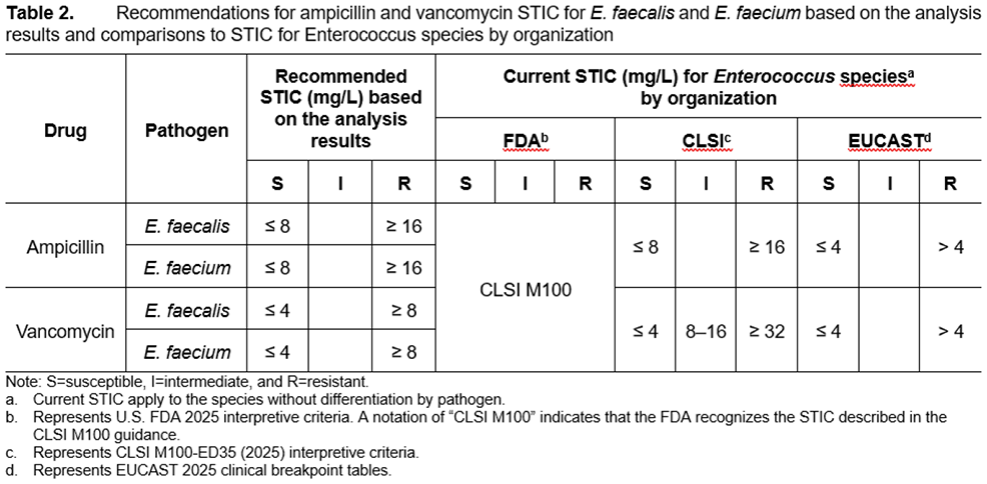

**Results:**

Percent probabilities of PK-PD target attainment by MIC on Days 1 to 2 and 5 to 6 for ampicillin and vancomycin are shown in Figure 1 and Figure 2, respectively. Table 1 shows candidate susceptible breakpoints for *E. faecalis* and *E. faecium* based on these data, with recommended and existing STIC shown in Table 2. These data suggest ampicillin and vancomycin susceptible breakpoints of ≤ 8 and ≤ 4 mg/L for both species, respectively, consistent with CLSI STIC. In totality, the results from the literature search for clinical data did not provide meaningful information.

**Conclusion:**

The results of these analyses provide support for recommendations for ampicillin and vancomycin STIC for *E. faecalis* and *E. faecalis.*

**Disclosures:**

All Authors: No reported disclosures

